# The efficacy and safety of Health Qigong for ankylosing spondylitis: Protocol for a systematic review and meta-analysis

**DOI:** 10.1097/MD.0000000000018734

**Published:** 2020-01-17

**Authors:** Biyuan Liu, Zhu Fan, Zheyi Wang, Man Li, Tao Lu

**Affiliations:** a School of Life Sciences, Beijing University of Chinese Medicine, Beijing, China; b School of Medicine, University of St Andrews, Scotland, UK.

**Keywords:** ankylosing spondylitis, Health Qigong, protocol, systematic review

## Abstract

**Background:**

Non-pharmacological treatments (education, exercise, and physical therapy) are remain basic approaches to long-term management of ankylosing spondylitis (AS) patients. As an important part of non-pharmacological treatments, Health Qigong is widely used for AS treatment. We will perform the systematic review to confirm the safety and efficacy of Health Qigong for AS.

**Methods:**

Systematical search of 6 electronic databases will be done, including English and Chinese, until December 2019. All randomized controlled trials (RCTs) involving Health Qigong in combination with conventional therapy for AS will be included. Study selection, data extraction, and validation were performed independently by 2 reviewers. RevMan (V.5.3) will be used for mata-analysis.

**Results:**

This systematic review will identify the safety and efficacy of Health Qigong in the treatment of AS and update evidence summaries of Health Qigong. At the end of the treatment, the primary outcome is Bath Ankylosing Spondylitis Disease Activity Index (BASDAI) with a range of 0 to 10,and the secondary outcomes will include functional ability that measured by Bath Ankylosing Spondylitis Functional Index (BASFI), mobility measured by Bath Ankylosing Spondylitis Metrology Index (BASMI), chest expansion, night spinal pain, adverse reactions, laboratory measures such as erythrocyte sedimentation rate (ESR) and C protein response (CRP).

**Conclusion:**

This study will provide evidence that whether Health Qigong can benefit patients with ankylosing spondylitis by reducing disease activity, alleviating pain to support the application of Health Qigong in the AS treatment.

**Registration number:**

CRD42019159126

## 1 Introduction

Ankylosing spondylitis (AS) is a chronic progressive inflammatory rheumatic disease with unknown etiology. And the incidence in Asia is 1.67/1000.^[1,2]^ AS is characterized clinically by inflammation of the axial skeleton, with early involvement of the Sacroiliac joints which have symptoms include back pain, spinal rigidity, as well as inflammation of the peripheral joints, shoulders, hips, and fingers/toes.^[3–5]^ In end stage, the pathological changes lead to fibrosis and calcification, resulting in fusion of the spine and loss of flexibility, similar as “bamboo” with a fastened securely position. Moreover years of pain and rigid deformation of spine eventually incur severe disability and economic cost.^[6]^

At present, the pathogenesis of AS has not been completely understood, that develops through complex interplay between genetic background and environmental factors.^[7,8]^ As we all known, AS is an autoimmune disease.^[9,10]^ Nonsteroidal anti-inflammatory drug (NSAID) treatment and physical therapy are important approaches for long-term treatment of patients with AS. NSAIDs are first-line therapy for symptomatic AS patients. Recent studies suggest that the regular NSAID use in AS make radiographic progression much slower than on-demand use.^[11]^ Furthermore, tumour necrosis factor inhibitor therapy improved life quality of more than two-thirds of AS patients who responded inadequately to NSAIDs.^[8]^

On the basis of reducing inflammation, finding alternative therapies is important for AS patients to alleviate symptoms and improve quality of life.^[12]^ Swimming has been confirmed as a suitable exercise, that produced better improvement in pain score and quality of life of the patients with AS.^[13,14]^ Health Qigong, including Tai Ji, Baduanjin (“Eight Section Brocade Qigong”), Wuqinxi (“Five Animals exercise”), Yijinjing and Liuzijue, are forms of traditional Chinese exercise, that are considered to not only increase body flexibility but also regulate mind. Previous studies have suggested that Health Qigong has great benefits in patients with various diseases, including back pain, metabolic diseases, cardiovascular diseases, cancer, and mental condition problems.^[15–19]^ Due to these advantages of Health Qigong, practitioners recommend using Qigong to improve clinical symptoms of AS patients.^[20]^

Even though the application of Health Qigong in AS treatment is increasing, systematic reviews and meta-analysis of Health Qigong for AS are rare.^[21]^ Hence, we will perform a systematic review of randomized clinical trials summarizing the existing evidence and assessing the effectiveness and safety of Health Qigong for AS.

## 2 Methods

This protocol has been registered with the International Prospective Register of Systematic Reviews (PROSPERO) (registration number: CRD42019159126; http://www.crd.york.ac.uk/PROSPERO) on November 2019. This review will adhered to Preferred Reporting Items for Systematic reviews and Meta-Analyses (PRISMA) to perform this study.^[22]^

### 2.1 Inclusion criteria

#### 2.1.1 Type of studies

All published clinical randomized controlled trials (RCTs) will be included. Cross-sectional studies, quasi-RCT, animal experiments, case observation reports, review articles, and other studies that are repeatedly published will be excluded. And the studies that the outcomes are not relevant to AS, Western medicine/traditional Chinese medicine is not used alone to the control group or other therapies (acupuncture, herbal dressing, or fumigant, etc) added on the experimental group will be excluded.

#### 2.1.2 Types of participants

Participants of both sexes and all age group with AS diagnosed according to the New York standard revised by the American College of Rheumatology in 1984 will be included in this review.

#### 2.1.3 Types of interventions

RCTs which involved Health Qigong alone, or in combination with Western/Traditional Chinese medicine, as well as those that can measure the therapeutic effect of Health Qigong will be included. Western medicine or traditional Chinese medicine alone will be selected as the control interventions.

#### 2.1.4 Types of outcome measures

The primary outcome is Bath ankylosing spondylitis disease activity index (BASDAI) with a range of 0 to 10.^[23]^ The secondary outcomes will include functional ability that measured by the Bath Ankylosing Spondylitis Functional Index (BASFI),^[24]^ mobility that measured by the Bath Ankylosing Spondylitis Metrology Index (BASMI),^[23]^ Chest expansion, nocturnal spinal pain, adverse reactions, and laboratory indicators, such as erythrocyte sedimentation rate (ESR) and C protein response (CRP).

### 2.2 Search strategy

We will perform a search of the following 6 databases for the period from inception to December 2019. English databases including PubMed, Embase, Cochrane Library, and Chinese databases including CNKI, Wanfang, and China Biology Medicine (CBM), using the terms “Ankylosing Spondylitis, AS” and “Health Qigong, Qigong, Baduanjin, Wuqinxi, Yijinjing, Tai Ji, Liuzijue, Ch’i Kung, Eight brocade, Five animals exercise, Six character formula.”

The search strategy for selecting the fields of topic, title, or abstract was unique referring to the characteristics of databases. The complete PubMed search strategy is in Table 1 and will be modified and used in other electronic databases.

**Table 1:**
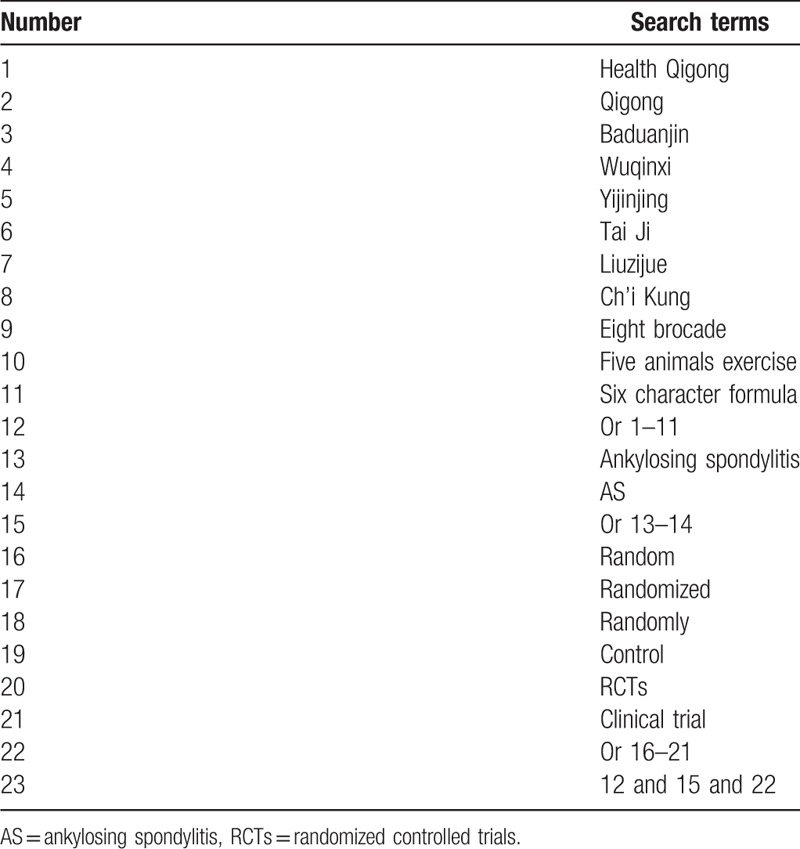
PubMed search strategy.

### 2.3 Data collection and analysis

#### 2.3.1 Study Selection

EndNote X8 will be used for literature management. The literature screening was conducted independently by 2 investigators (BYL and ZF), and the differences were determined through discussion within the group. After excluding the apparently unrelated literature, read the title and abstract for further evaluation, then obtain the full potential research and re-evaluate.

**Figure 1:**
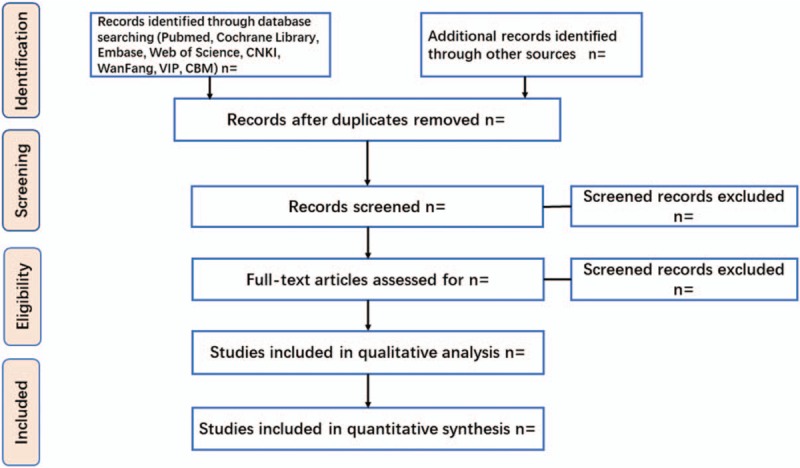
The PRISMA flow char of study. PRISMA = Preferred Reporting Items for Systematic reviews and Meta-Analyses.

The details of the selection process are shown as the PRISMA flow char of study (Fig. 1).

#### 2.3.2 Data extraction

Two investigators (BYL and ZF) extract from the included trials independently, and enter the data into a structured characteristics table, which includes the following aspects: basic general information of the literature, such as author, publication time, country, etc; baseline data of intervention group and control group; methodological quality of included study; intervention method of intervention group and control group; outcome index. If necessary, contact the author for more information. Disagreements between the 2 investigators can be resolved through discussion, and a third investigator (ML) who is consulted for expert opinions.

#### 2.3.3 Quality assessment

Quality assessment was performed in accordance with the Cochrane Intervention System Review Manual (version 5.2.0) including random sequence generation, allocation concealment, blinding of participants and personnel, blinding of outcome assessment, incomplete outcome data, selective reporting and other sources of bias, a total of 7 items. The evaluation results are expressed as “high risk,” “unclear,” and “low risk.” Among them, “high risk” means that the implementation method is wrong, “low risk” means correct, and “unclear” means that the research lacks description of this part. The main outcomes quality will also be assessed with the GRADE approach. Two investigators (BYL and ZF) will perform the evaluation of quality independently, if the result is discrepancies, it will be decided after discussion with another investigators (ML).

#### 2.3.4 Data analysis

According to the basic characteristics of the included studies, the Meta analysis will be performed using Review Manager version 5.3 provided by the Cochrane Collaboration. With *P* = .1 as the test level, the presence of heterogeneity between studies will be tested. If there is heterogeneity, a fixed effect model is used when *P* > .1, and a random effect model is used when *P* < .1.

The categorical variable selects the odds ratio (OR) as the effect quantity, and the continuous variable selects the weighted mean difference (WMD) or the standardized mean difference (SMD) as the effect quantity, with *P* = .05 as the test standard; for individual studies with special interventions and statistical indicators, only the effect size and 95% confidence interval are calculated, and then descriptive analysis is performed. If necessary, use a funnel plot to explore the publication bias, and use a sensitive I-analysis to test the stability of the results when the bias is significant.

#### 2.3.5 Subgroup analysis

If the heterogeneity is obvious, subgroup analysis will be carried out according to the characteristics of the study for exploration of potential sources of heterogeneity. The criteria for a subgroup analysis are as follows:

1. Type of control interventions.
2. Type of Health Qigong.
3. Exercise frequency and duration.

#### 2.3.6 Sensitivity analysis

For the quality analysis, we will investigate the influence of bias on results by undertaking a sensitivity analysis of the primary outcomes and excluding the high risk of bias studies.

### 2.4 Ethics and dissemination

Ethics clearance will not be required as this is a secondary analysis of published data and no patients will be involved in this study.

## 3 Discussion

Arthritis and pain caused by ankylosing spondylitis (AS) severely affect all aspects of patients’ daily life.^[25]^ Therefore, it is necessary to find effective non-drug therapies to alleviate the great physical and psychological suffering of AS patients. Health Qigong, as a traditional Chinese sport, has a significant effect on improving immune function, anti-inflammatory, and relieving back pain.^[20,26,27]^ Previous searches found that Health Qigong as a complementary and alternative non-drug treatment in China has been widely used in the treatment of ankylosing spondylitis.^[28]^ However, there is still no systematic review clearly demonstrating the role of Health Qigong in the treatment of ankylosing spondylitis.

The results of this study will confirm whether Health Qigong can reduce the clinical symptoms and related laboratory indicators of patients with AS, which will give an evidence for Health Qigong in clinical applications. The small number of related literature and the high heterogeneity of different Qigong types used in the research may bring limitations to this study. If there is enough randomized controlled double-blind experimental research, we will perform a subgroup analysis to determine specific qigong programs and exercise time that are more beneficial for ankylosing spondylitis patients. In addition, qigong is widely spread in Asia, especially China, but it is not well accepted in other parts of the world, so publication bias will also be considered.

In conclusion, this study will investigate the effectiveness of Health Qigong as a treatment of ankylosing spondylitis.

## Author contributions

**Data curation:** Biyuan Liu, Zhu Fan, Man Li.

**Formal analysis:** Man Li, Tao Lu.

**Methodology:** Biyuan Liu, Zhu Fan.

**Project administration:** Tao Lu.

**Software:** Zheyi Wang.

**Supervision:** Tao Lu

**Validation:** Tao Lu.

**Visualization:** Biyuan Liu, Zheyi Wang.

**Writing – original draft:** Biyuan Liu, Zhu Fan.

**Writing – review & editing:** Tao Lu.

Tao Lu orcid: 0000-0002-8247-8387.
